# Supplementary material for: SELENOF Controls Proliferation and Cell Death in Breast-Derived Immortalized and Cancer Cells
Source: Cancers (Basel). 2023 Jul 19;15(14):3671. doi: 10.3390/cancers15143671 (PMC10377602; doi:10.3390/cancers15143671)
Supplement: Supplementary file 1 [file cancers-15-03671-s001.zip › cancers-2464369-supplementary caption.pdf]

Supplementary information

Figure S1. Proliferation of MCF-10A wild type versus SELENOF knockout cells. (A) SELENOF levels in whole cell extracts were determined by western blotting of MCF-10A wild type (WT) CRISPR-Cas9 non-targeting sgRNA control cells or single-cell derived lines from SELENOF knockout (KO) cells. β-Actin is shown as a loading control. (B) Cell proliferation was measured over 6 days in culture of cells from (A) including naïve MCF-10A cells. Cells were fixed, stained with crystal violet, solubilized in 1% SDS, and quantified as absorbance at 570nm.

Figure S2. Silencing SELENOF increases Ki67 mRNA in breast cancer lines. HCC70 and MDA-MB-157 cells were transfected with siNeg or siSELENOF, 20nM each, for 48 hours. Total RNA was isolated using the TRIZOL method and gene expression for SELENOF or MKI67 was determined by RT-QPCR. * $p<0.05$, **** $p<0.0001$.

Figure S3. The expression levels of SELENOF, p21 and p27 in 3D acini. The levels of SELENOF, p21 and p27 were examined by western blotting of whole cell extracts from 3D acini of MCF-10A WT or SELENOF KO cells grown in 5% matrigel for 20 days. β-Actin is shown as a loading control.

Figure S4. Gene expression was measure by RT-QPCR to determine the silencing efficiency of p21 and p27 siRNAs transfected in MCF-7 SELENOF cells, 10nM each, for 48 hours.

Figure S5. MCF-7 SELENOF cell viability upon SELENOF induction. Cells were treated with vehicle control (curve in black) or with Dox 1μg/ml. In one cohort Dox was withdrawn after 3

days (curve in red); in another cohort Dox was withdrawn after 6 days (curve in blue), in the last cohort Dox was given continuously (curve in green). Medium was changed and Dox was replenished every 48 hours. Cell viability was determined by crystal violet staining.

Figure S6. Quantification of DNA fragmentation (A) and phagosome formation (B) in MCF-7 SELENOF cells from Figure 6.

Figure S7. Ferroptosis does not contribute to SELENOF-induced cell death. Cell viability was determined by crystal violet staining of cells treated with -/+ 1µg/ml Dox and -/+1µM ferrostatin-1 (Fer1) which could not rescue from cell death. ML210 (3µM), a GPX4 inhibitor was used as a positive control to induce ferroptosis. Data was normalized to vehicle control and shown as 100%. *ns* non significant, * $p<0.05$.
