# Supplementary material for: SELENOF Controls Proliferation and Cell Death in Breast-Derived Immortalized and Cancer Cells
Source: Cancers (Basel). 2023 Jul 19;15(14):3671. doi: 10.3390/cancers15143671 (PMC10377602; doi:10.3390/cancers15143671)
Supplement: Supplementary file 1 [file cancers-15-03671-s001.zip › cancers-2464369-supplementary figures.pdf]

Figure S1.

A.

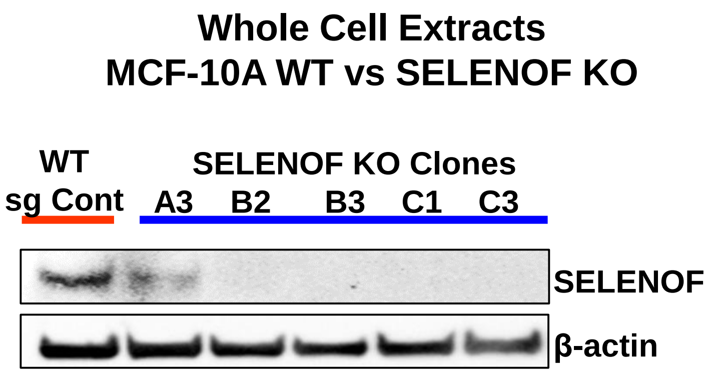
Whole Cell Extracts
MCF-10A WT vs SELENOF KO
WT
sg Cont
SELENOF KO Clones
A3
B2
B3
C1
C3
SELENOF
β-actin

B.

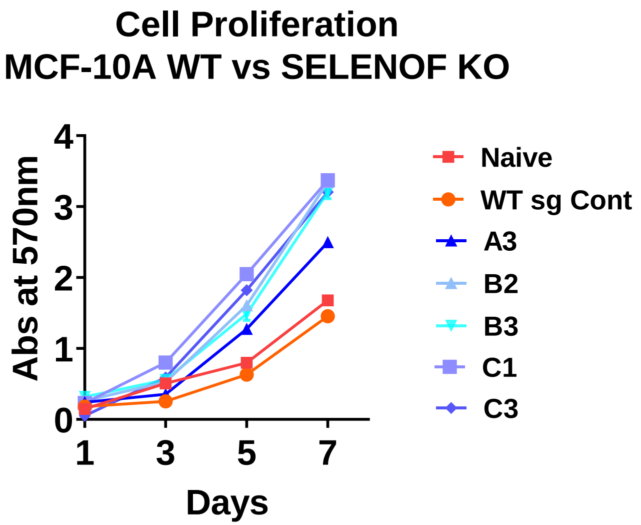
Cell Proliferation
MCF-10A WT vs SELENOF KO
Abs at 570nm
0
1
2
3
4
1
3
5
7
Days
Naive
WT sg Cont
A3
B2
B3
C1
C3

Figure S2.

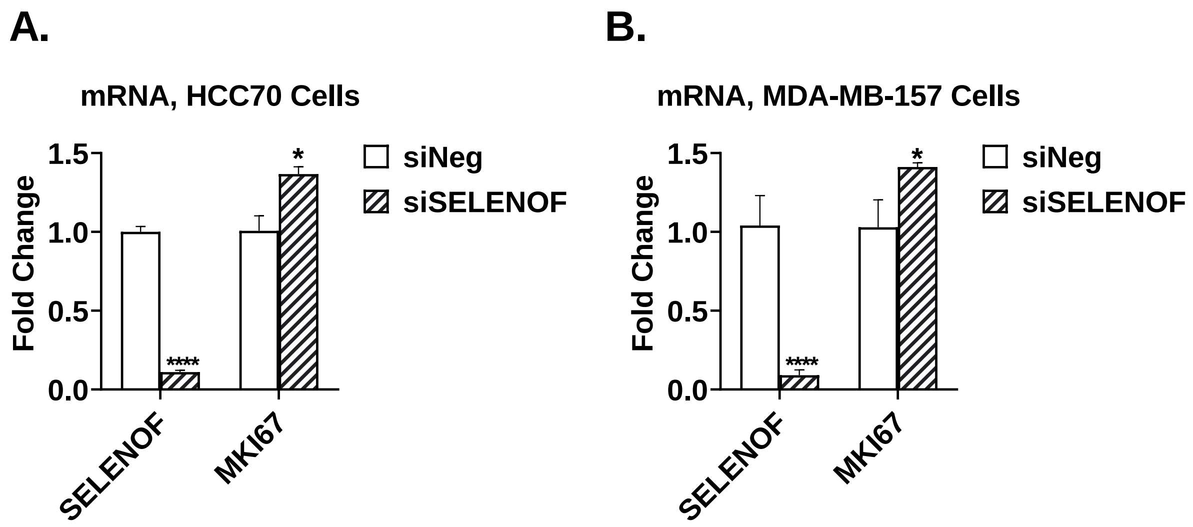
A.
mRNA, HCC70 Cells
Fold Change
1.5
1.0
0.5
0.0
****
*
SELENOF
MKI67
siNeg
siSELENOF
B.
mRNA, MDA-MB-157 Cells
Fold Change
1.5
1.0
0.5
0.0
****
*
SELENOF
MKI67
siNeg
siSELENOF

Figure S3.

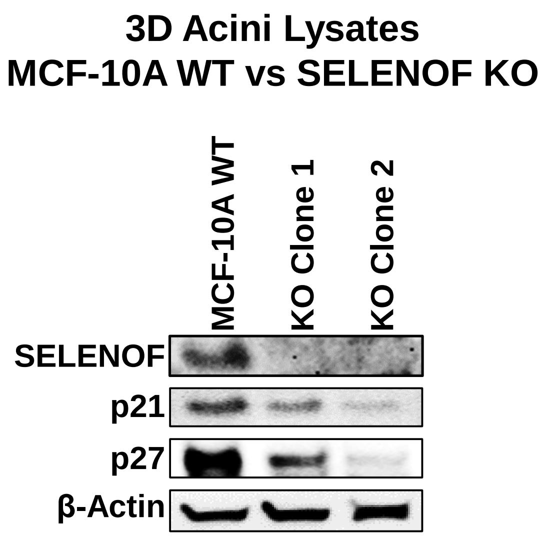
3D Acini Lysates
MCF-10A WT vs SELENOF KO
MCF-10A WT
KO Clone 1
KO Clone 2
SELENOF
p21
p27
β-Actin

Figure S4.

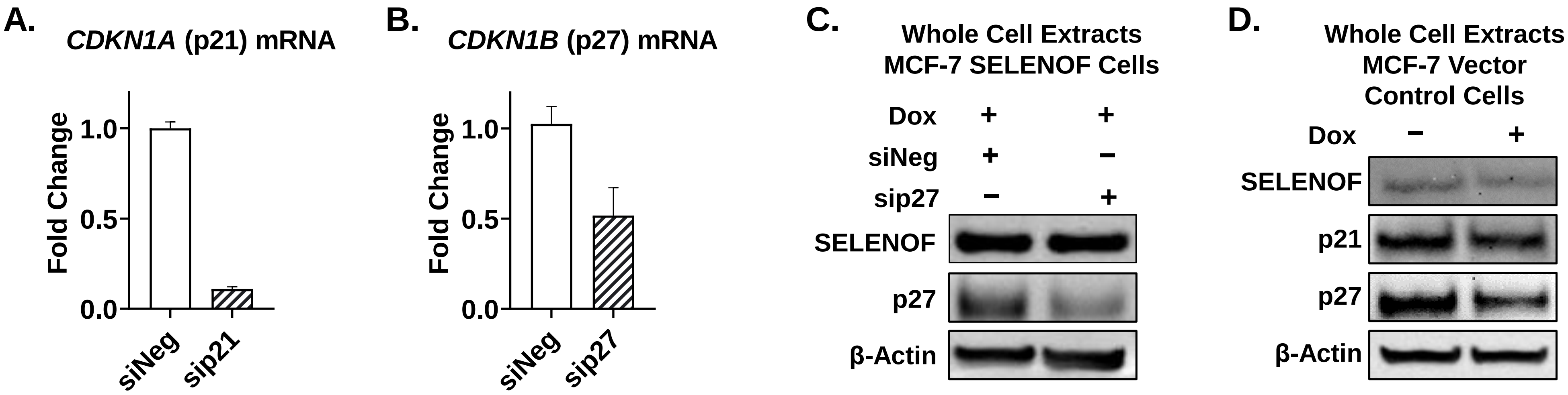
A.
CDKN1A (p21) mRNA
Fold Change
1.0
0.5
0.0
siNeg
sip21
B.
CDKN1B (p27) mRNA
Fold Change
1.0
0.5
0.0
siNeg
sip27
C.
Whole Cell Extracts
MCF-7 SELENOF Cells
Dox + +
siNeg + −
sip27 − +
SELENOF
p27
β-Actin
D.
Whole Cell Extracts
MCF-7 Vector
Control Cells
Dox − +
SELENOF
p21
p27
β-Actin

Figure S5.

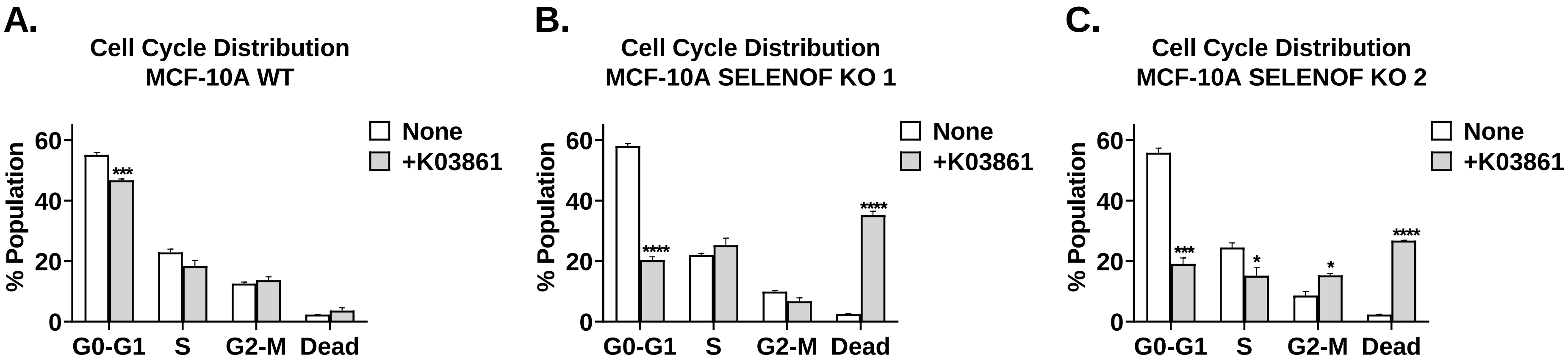
A.
Cell Cycle Distribution
MCF-10A WT
% Population
60
40
20
0
G0-G1
S
G2-M
Dead
***
None
+K03861
B.
Cell Cycle Distribution
MCF-10A SELENOF KO 1
% Population
****
****
None
+K03861
C.
Cell Cycle Distribution
MCF-10A SELENOF KO 2
% Population
***
*
*
****
None
+K03861

Figure S6.

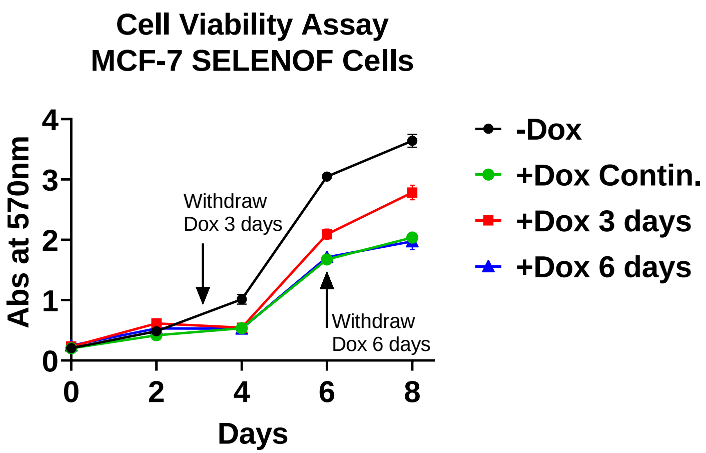

Cell Viability Assay
MCF-7 SELENOF Cells
Abs at 570nm
4
3
2
1
0
0
2
4
6
8
Days
Withdraw
Dox 3 days
Withdraw
Dox 6 days
-Dox
+Dox Contin.
+Dox 3 days
+Dox 6 days

Figure S7.

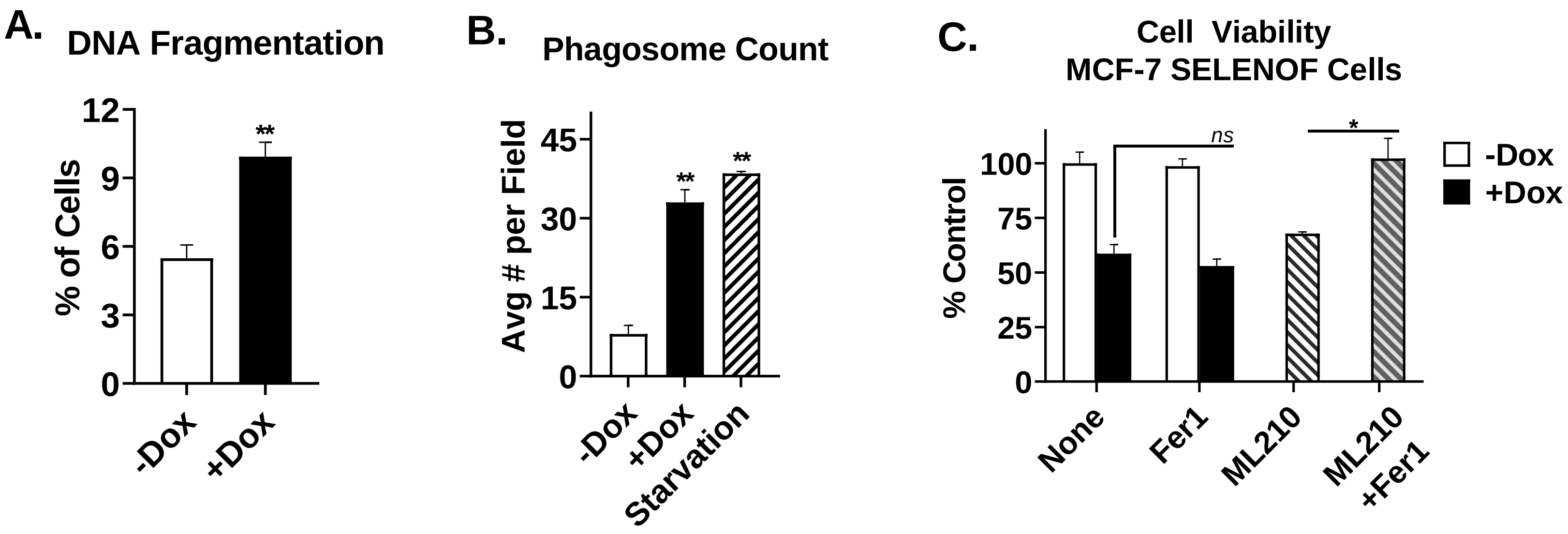
A.
DNA Fragmentation
% of Cells
0
3
6
9
12
**
-Dox
+Dox
B.
Phagosome Count
Avg # per Field
0
15
30
45
**
**
-Dox
+Dox
Starvation
C.
Cell Viability
MCF-7 SELENOF Cells
% Control
0
25
50
75
100
ns
*
None
Fer1
ML210
ML210
+Fer1
-Dox
+Dox
