# Supplementary material for: SELENOF Controls Proliferation and Cell Death in Breast-Derived Immortalized and Cancer Cells
Source: Cancers (Basel). 2023 Jul 19;15(14):3671. doi: 10.3390/cancers15143671 (PMC10377602; doi:10.3390/cancers15143671)
Supplement: Supplementary file 1 [file cancers-15-03671-s001.zip › cancers-2464369-supplementary File S1.pptx]

## Slide 1
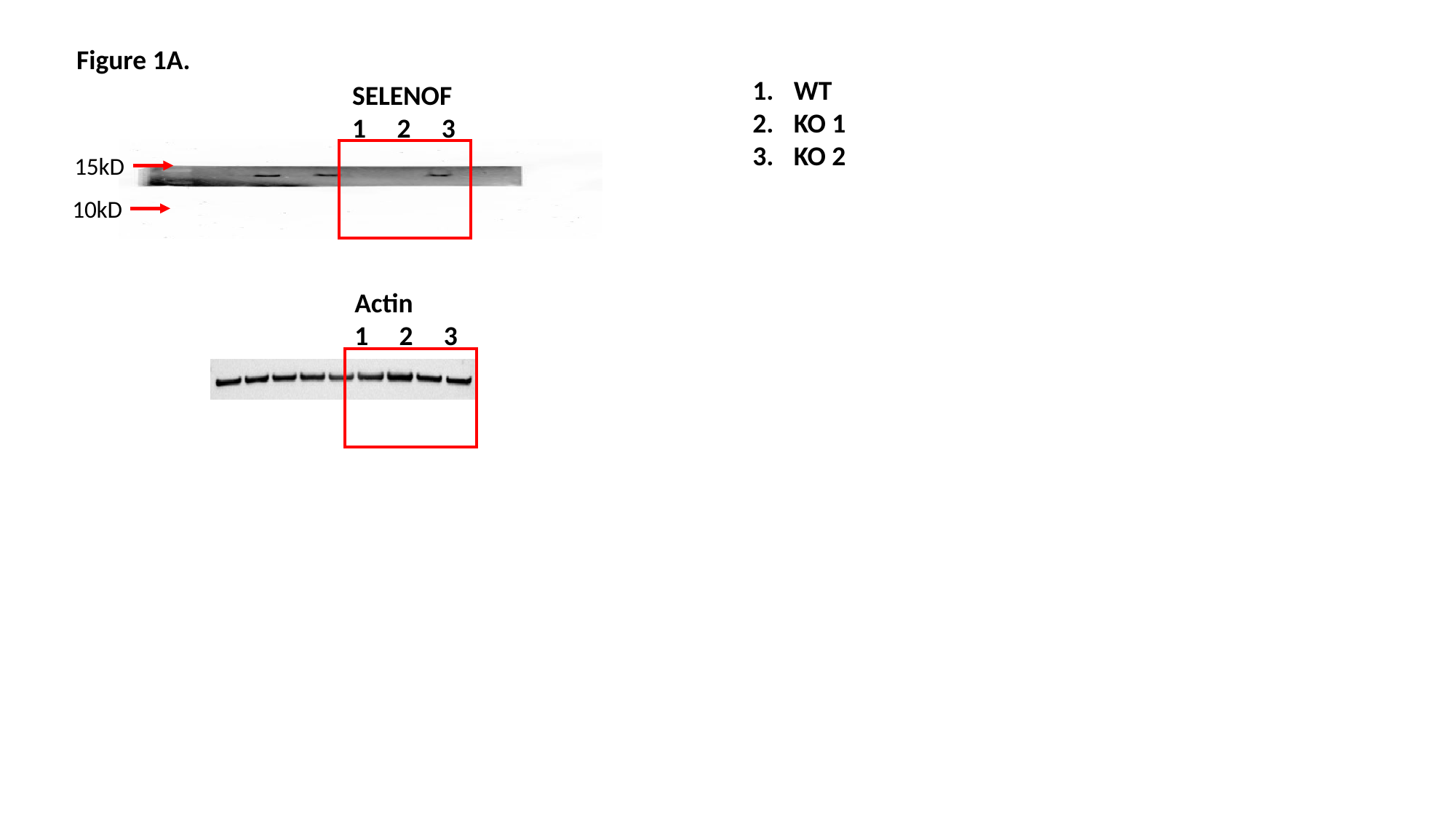

Figure 1A.
WT
KO 1
KO 2
SELENOF
1 2 3
15kD
10kD
Actin
1 2 3

## Slide 2
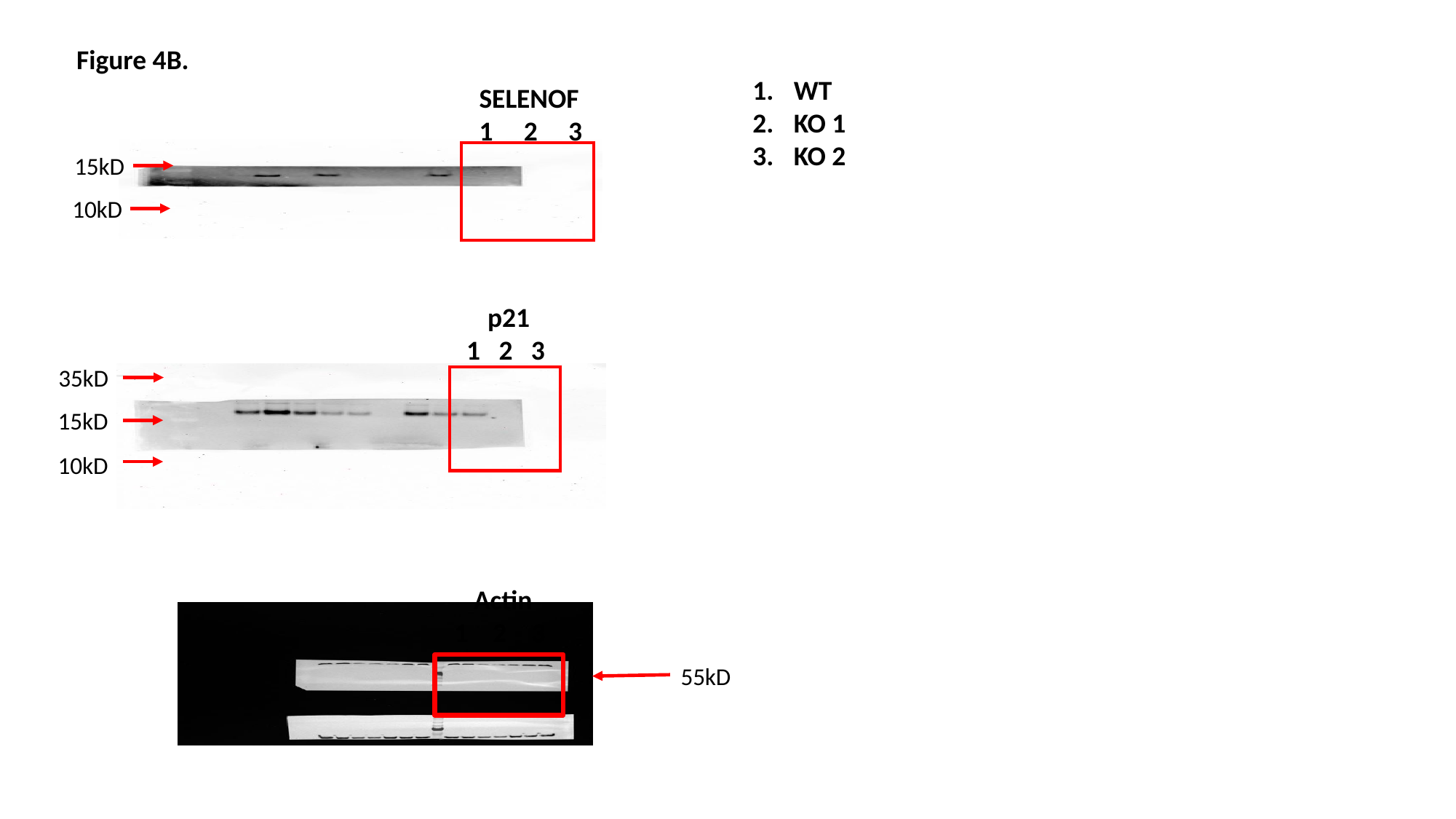

Figure 4B.
WT
KO 1
KO 2
SELENOF
1 2 3
15kD
10kD
p21
1 2 3
15kD
10kD
35kD
Actin
1 2 3
55kD

## Slide 3
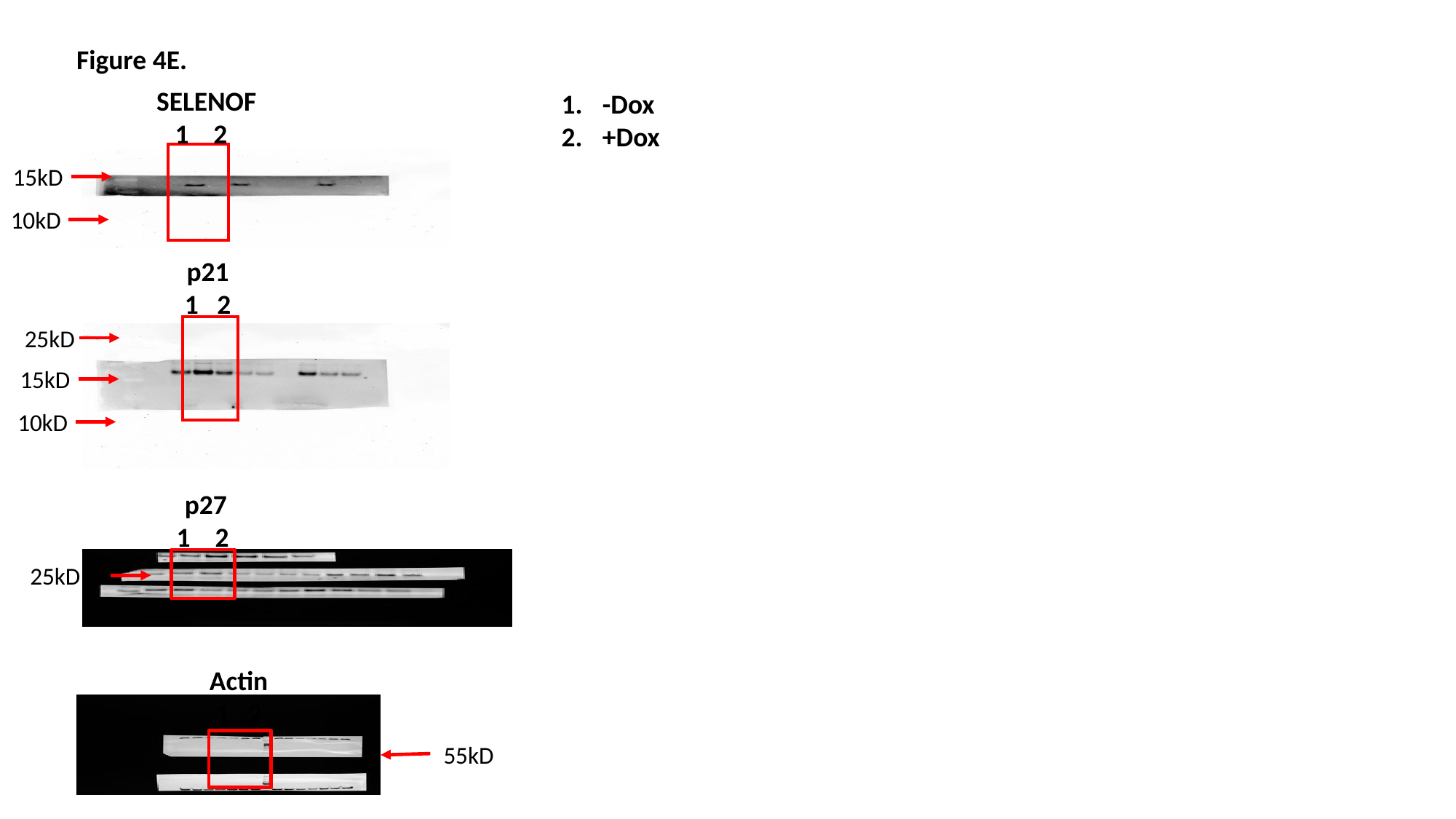

Figure 4E.
SELENOF
 1 2
15kD
10kD
-Dox
+Dox
p21
 1 2
25kD
15kD
10kD
p27
1 2
25kD
Actin
 1 2
55kD

## Slide 4
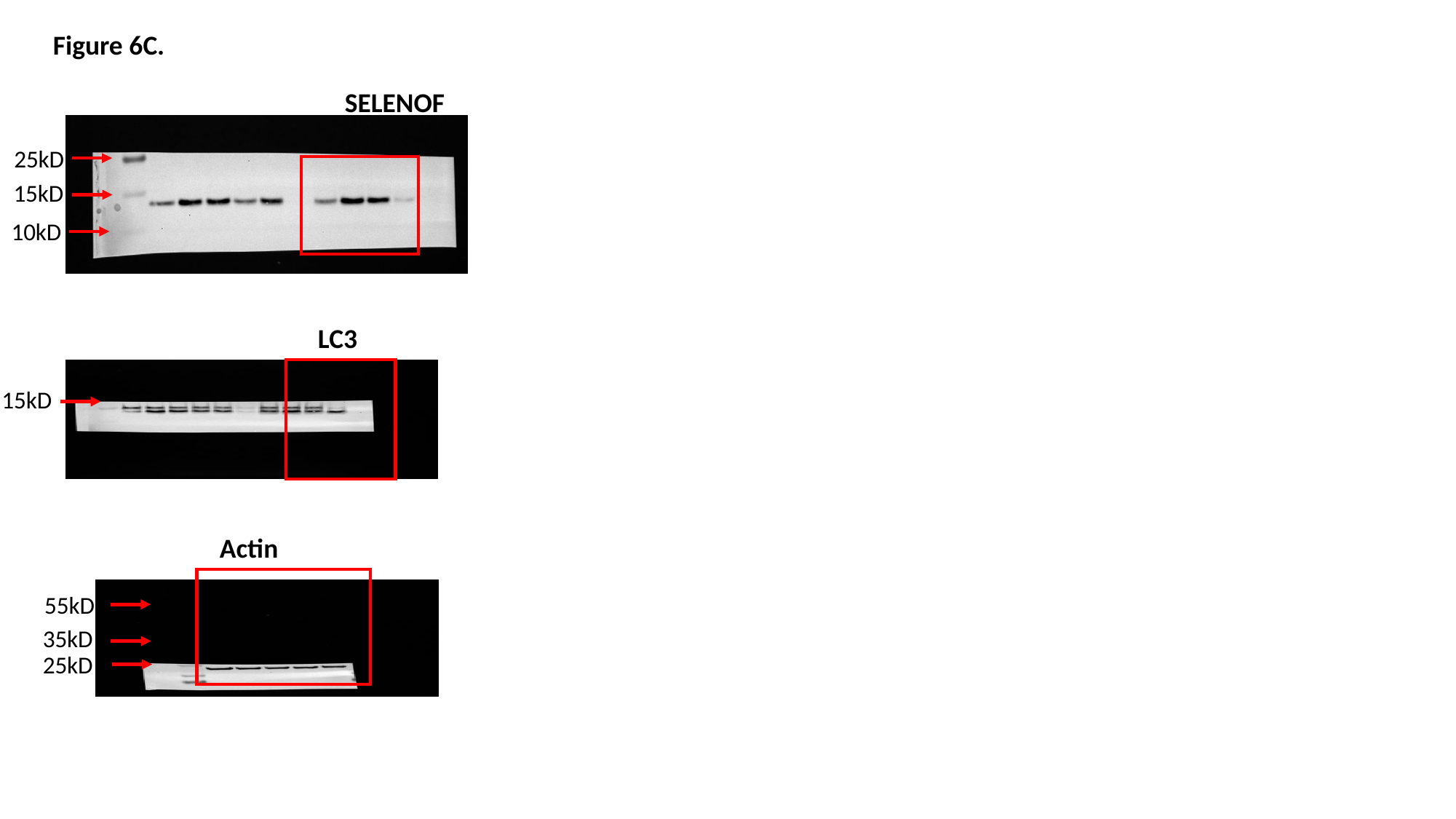

Figure 6C.
SELENOF
25kD
15kD
10kD
LC3
15kD
Actin
55kD
35kD
25kD
